# Evaluation of neuroprotective and immunomodulatory properties of mesenchymal stem cells in an ex vivo retinal explant model

**DOI:** 10.1186/s12974-022-02418-w

**Published:** 2022-03-02

**Authors:** Élodie Reboussin, Juliette Buffault, Françoise Brignole-Baudouin, Annabelle Réaux-Le Goazigo, Luisa Riancho, Céline Olmiere, José-Alain Sahel, Stéphane Mélik Parsadaniantz, Christophe Baudouin

**Affiliations:** 1 Sorbonne Université UM80, INSERM UMR 968, CNRS UMR 7210, Institut de la Vision, IHU FOReSIGHT, 17 rue Moreau, 75012 Paris, France; 2 Service 3, CHNO des Quinze-Vingts, INSERM-DGOS CIC 1423, 28 rue de Charenton, 75012 Paris, France; 3 Laboratoire, CHNO des Quinze-Vingts, 28 rue de Charenton, 75012 Paris, France; 4 Laboratoires THEA, Clermont-Ferrand, France; 5 Department of Ophthalmology, The University of Pittsburgh School of Medicine, Pittsburgh, PA 15213 USA

**Keywords:** Glaucoma, Neuroprotection, Immunomodulation, Cellular therapy, Mesenchymal stem cell, Microglia, Retinal ganglion cell

## Abstract

**Background:**

Glaucoma is a blinding degenerative neuropathy in which the death of retinal ganglion cells (RGCs) causes progressive loss of visual field and eventually vision. Neuroinflammation appears to be a key event in the progression and spread of this disease. Thus, microglial immunomodulation represents a promising therapeutic approach in which mesenchymal stem cells (MSCs) might play a crucial role. Their neuroprotective and regenerative potentials have already raised hope in animal models. Yet no definitive treatment has been developed, and some safety concerns have been reported in human trials. In the present study, we investigated the neuroprotective and immunomodulatory properties as well as the safety of MSCs in an ex vivo neuroretina explant model.

**Methods:**

Labeled rat bone marrow MSCs were placed in coculture with rat retinal explants after optic nerve axotomy. We analyzed the neuroprotective effect of MSCs on RGC survival by immunofluorescence using RBPMS, Brn3a, and NeuN markers. Gliosis and retinal microglial activation were measured by using GFAP, CD68, and ITGAM mRNA quantification and GFAP, CD68, and Iba1 immunofluorescence stainings. We also analyzed the mRNA expression of both ‘M1’ or *classically activated* state inflammatory cytokines (TNFα, IL1β, and IL6), and ‘M2’ or *alternatively activated* state microglial markers (Arginase 1, IL10, CD163, and TNFAIP6).

**Results:**

The number of RGCs was significantly higher in retinal explants cultured with MSCs compared to the control group at Day 7 following the optic nerve axotomy. Retinal explants cultured with MSCs showed a decrease in mRNA markers of gliosis and microglial activations, and immunostainings revealed that GFAP, Iba1, and CD68 were limited to the inner layers of the retina compared to controls in which microglial activation was observed throughout the retina. In addition, MSCs inhibited the M1 phenotype of the microglia. However, edema of the explants was observed in presence of MSCs, with an increase in fibronectin labeling at the surface of the explant corresponding to an epiretinal membrane-like phenotype.

**Conclusion:**

Using an ex vivo neuroretina model, we demonstrated a neuroprotective and immunomodulatory effect of MSCs on RGCs. Unfortunately, the presence of MSCs also led to explant edema and epiretinal membrane formation, as described in human trials. Using the MSC secretome might offer the beneficial effects of MSCs without their potential adverse effects, through paracrine signaling.

**Supplementary Information:**

The online version contains supplementary material available at 10.1186/s12974-022-02418-w.

## Background

Glaucoma is a blinding degenerative neuropathy in which retinal ganglion cell (RGC) death leads to a progressive loss of visual field and eventually vision [1]. Preventing RGC death through neuroprotective strategies could completely modify the visual prognosis of patients with glaucoma. In the last few decades, MSCs and their neuroprotective and regenerative properties have raised considerable hope in glaucoma therapy. However, no protocol has yet been validated in humans [2].

MSCs are multipotent cells found in many adult tissues, able to proliferate, self-renew and differentiate into many types of specialized cells, including osteocytes, adipocytes, or chondrocytes. Moreover, MSCs produce numerous growth or neurotrophic factors, leading to the hypothesis that they could play a major role in stimulating the survival and growth of RGCs [2–4]. MSC-based therapies in retinal diseases have been explored in order to prevent or delay RGCs death and even to regenerate RGCs [5, 6]. Intravitreal transplantation of bone marrow MSCs (BMMSCs) in rat ocular hypertension models has been shown to be highly neuroprotective by reducing RGCs death [3, 4, 7]. Anterior chamber transplantation of rat BMMSCs in a rat ocular hypertension model also demonstrated neuroprotective effects as measured by peripheral RGC density and a significant but transient reduction in IOP [8].

In glaucoma patients as well as in rodent preclinical models of glaucoma, higher expression of glial fibrillary acidic protein (GFAP) was found to be associated with the degeneration of the optic nerve fibers [9]. So, glial immunomodulation represents a promising therapeutic approach in which MSCs might play a crucial role. Indeed, in glaucoma, RGC loss is associated with an inflammatory process caused by activation of resident glial cells, e.g., microglial cells, Müller cells, and astrocytes [9–13]. Once activated, these cells release a cocktail of cytokines, chemokines, and reactive oxygen species (ROS) and consequently contribute to RGCs loss. Among these cells, microglial cells, also called sentinel immune cells, exist in two dynamic and opposite activated states with neurotoxic or neuroprotective effects [14–16]. During retinal degeneration, microglia are activated and particularly polarized to a pro-inflammatory M1 phenotype [17]. The “classical activation state” or “M1 state” is characterized by the production of ROS and secretion of numerous pro-inflammatory molecules such as TNFα, IL-1β, and IL-6. The second state, also known as the “alternatively activated state” or “M2 state,” is induced by IL-4 or IL-13. It allows clearance of debris, restores tissue homeostasis, and promotes tissue repair by inhibiting inflammation through the production of anti-inflammatory, neurotrophic factors, and chemokine receptors [14–16, 18]. Several M2 phenotype markers characterize this M2 state, e.g., the enzyme Arginase 1 (ARG1), a marker of microglia involved in tissue repair and phagocytosis, the receptor CD163, a marker of microglia implicated in the anti-inflammatory process and healing, IL-10, an anti-inflammatory cytokine used by the M2 subtype to antagonize the pro-inflammatory phase and healing, and TNFα-stimulated gene-6 (TSG-6/TNFAIP6), which is a key anti-inflammatory factor produced by MSCs [19–22]. Reducing the pro-inflammatory M1 phenotype or inducing M2 microglial polarization might represent a potential and promising therapeutic option to treat neuroinflammatory degenerative diseases such as glaucoma [9, 23–25]. Among potent immunomodulators of microglial polarization, MSCs possess potent immunoregulatory properties and might inhibit a harmful inflammatory reaction in the diseased retina [2, 25, 26].

Obtaining a model of RGC loss to reproduce the degenerative process observed in glaucoma is a challenge. Indeed, many animal models have been developed, but have encountered reproducibility issues in the rate of RGC loss [2]. The retinal explant model offers the advantage compared to cell lines or dissociated cultures, to maintain an in vivo-like architecture with all neuroretina layers retaining intercellular interactions; it allows direct access to the RGC layer; it limits the number of animals used and is less time-consuming than the classical use of animal models, considering the degeneration rate of RGCs. Optic nerve transection leads to the death by apoptosis of 90% of injured RGCs within 14-day post-axotomy in vivo [27–29]. Similar to these results, RGC loss by apoptosis was shown in retinal explants [30–32]. Thus, this model fills the gap between relevant but time/cost/animal-consuming preclinical models and rapid/high-throughput cell culture models often based on one single cell type, which cannot substitute for the complexity of an entire tissue.

Considering the promising MSC neuroprotective results previously demonstrated in animal models, our objective was to evaluate the effects of MSCs use as a neuroprotective and immunomodulatory therapy in a neuroretina explant model of RGC degeneration, with a particular attention to immuno-inflammatory patterns and potential safety issues.

## Materials and methods

### Animals

Adult (6–8 weeks old) male Long Evans rats weighing 250–300 g were purchased from Janvier Laboratories and used for harvesting fresh BMMSCs and retinal explants to proceed to coculture. Animals were kept in pathogen-free conditions with food and water ad libitum and housed in a 12-h light/12-h dark cycle. All experiments were conducted after evaluation and approval by the Institutional Animal Care and Use Committee following the guidelines from Directive 2010/63/EU of the European Parliament on the protection of animals used for scientific purposes. All experimental procedures were approved by the local animal care ethics committee C2EA-05—Charles Darwin.

### MSC isolation and culture

Fresh BMMSCs were harvested from femurs of 6-week-old Long Evans rats (Janvier Laboratories). Briefly, femurs were isolated, and the cavities were flushed with 5 ml of expansion medium composed of αMEM (Thermo Fisher Scientific, ref. 31095029), 10% heat-inactivated fetal calf serum (Thermo Fisher Scientific, ref. 10499044), and 1% penicillin–streptomycin 10,000 U/ml (Thermo Fisher Scientific, ref. 15140122), through a 21G needle. Cells were incubated in 75-cm^2^ flasks (200,000 cells/cm^2^) at 37 °C in 5% CO_2_ humidified air for 72 h. Three days later, nonadherent cells were washed away with DPBS (Thermo Fisher Scientific, ref. 14190169), and fresh medium was added and kept until the first passage. MSCs were cultured with a weekly passage at a seeding rate of 100,000 cells/ml and characterized using flow cytometry (FCM) until cocultured with retinal explants.

### Flow cytometry

BMMSCs were cultured in 75-cm^2^ flasks until near confluence, in standard culture conditions (5% CO_2_, 37 °C, and saturated humidity atmosphere). Then, MSCs were harvested using Trypsin 0.05% EDTA (Thermo Fisher Scientific, ref. 25300054), washed twice with DPBS, and suspended in DPBS at 500,000 cells/ml after numeration using Flow-Count fluorospheres (Beckman Coulter, ref. 7547053). The suspension of live MSCs was characterized using flow cytometry (Cytomics FC 500 flow cytometer, Beckman Coulter, Miami, FL, USA) and positive (CD29, CD90, CD73) and negative (CD11b/c, CD45, CD68, MHC class II (Ia)) antigens were analyzed. The antibodies used for this characterization are presented in Table 1. Incubations for direct fluorochrome-conjugated antibodies and for non-conjugated primary antibodies were performed in 50 µl DPBS for 30 min in the dark. For indirect immunostaining, another 30-min incubation was carried out with the secondary antibody. After immunostaining, cells were washed once in DPBS and finally suspended in 300 µl of DPBS for flow cytometric acquisition.

**Table 1:** Antibodies used for the MSC characterization using flow cytometry

Antibody	Host	Supplier	Reference
Positive markers			
CD29	Armenian hamster	eBioscience	11-0291-80
CD73	Mouse	BD Biosciences	551123
CD90	Mouse	eBioscience	45-0900-80
Negative markers			
CD11b/c	Rabbit	BD Pharmingen	554862
CD45	Mouse	Serotec	MCA43PE
CD68	Mouse	Serotec	MCA341R
Ia	Mouse	Bio-Rad	MCA46G
Isotype control FITC	Armenian hamster	eBioscience	11-4888-81
IgG2a kappa Isotype control	Mouse	eBioscience	45-4724-80
IgG1, κ	Mouse	BD Pharmingen	554680
IgG-UNLB	Mouse	SouthernBiotech	0107-01

### Retinal explant cultures

Eight-week-old Long Evans rats (Janvier Laboratories) were used to collect retinal explants for culture, as previously described [30, 33]. Rats were euthanized, and the eyes were excised, then quickly placed in an ice-cold CO_2_ independent medium (Thermo Fisher Scientific, ref. 18045-054). Under sterile conditions at 4 °C in a CO_2_ independent medium, the anterior chamber, lens, and vitreous body were removed, and the retina was separated from the surrounding ocular tissues by dissection with curved microforceps. Retinas were then cut into four equal quarters and flat-mounted with the RGC layer up on Millicell-Polytetrafluoroethylene (PTFE) 0.4-μm culture plate inserts (Merck Millipore, ref. PICM01250), in culture medium composed of Neurobasal A (Thermo Fisher Scientific, ref. 10888022), 2% B27 supplement (Thermo Fisher Scientific, ref. 0080085-SA), 1% N2 supplement (Thermo Fisher Scientific, ref. 17502048), l-glutamine (Thermo Fisher Scientific, ref. 25030032), and 1% penicillin–streptomycin 10,000 U/ml, at 37 °C in 5% CO_2_ humidified air. The next day, half of the medium was changed, which was then changed every 48 h thereafter.

### Pharmacologic agents

Brain-derived neurotrophic factor (BDNF) (Sigma-Aldrich, ref. SRP3014) or *N*-methyl-d-aspartate (NMDA) (Sigma-Aldrich, ref. M3262) were diluted daily in retinal culture medium and added in direct contact with the RGCs at a concentration of 200 ng/ml or 50 µM, respectively, in a 3-µl droplet carefully dispensed onto the surface of the explant [30]. Also, half of the retinal culture medium containing BDNF or NMDA was changed at Day 1 and every 48 h thereafter.

### Retinal explant–MSC coculture

MSCs from passages 5 to 7 were used for this experiment. Before coculture, MSCs were trypsinized, washed in DPBS, and suspended in retinal explant culture medium at 5000 cells/µl [30, 33]. MSCs were labeled with Vybrant™ DiO Cell-Labeling Solution (Invitrogen, V22886) before the coculture to track them within the coculture system. Once the retinal explants were flat-mounted with the RGC layer up, 2 µl of this MSC suspension was gently dispensed onto the surface of the retinal explants.

### Immunohistochemistry

#### Tissue preparation

For cryosections, retinal explants were fixed in PFA 4% for 24 h at 4 °C, then dehydrated in sucrose 30% (DPBS; pH 7.4) overnight at 4 °C, before being embedded in OCT (Tissue-Tek® O.C.T. Compound, Sakura® Finetek) and frozen. Cryosections of retinal explants 12 µm in thickness were performed using a Leica cryostat CM 3050S and stored at − 20 °C until use. For wholemount counting, retinal explants were fixed in PFA at 4 °C overnight and rinsed in DPBS before the immunofluorescence step.

#### Dual immunofluorescence labeling in whole flat-mounted retinal explants or cryosections

Retinal explant wholemounts or cryosections were incubated for 2 h at room temperature (RT) in a blocking buffer (5% BSA, 2% Triton X-100, and 0.5% Tween20, in DPBS) and left to incubate overnight at 4 °C in the incubation buffer (2.5% BSA, 1% Triton X-100 and 0.25% Tween20, in DPBS) with polyclonal rabbit anti-RBPMS (1/200, Merck Millipore, ref. ABN 1362), monoclonal mouse anti-Brn-3a (1/100, Merck Millipore, ref. MAB1585), monoclonal mouse anti-NeuN (1/500, Merck Millipore, ref. MAB377), monoclonal mouse anti-CD68 (1/400, AbD Serotec, ref. MCA341R), polyclonal rabbit anti-GFAP (1/500, Dako, Agilent, ref. Z033429-2), polyclonal rabbit anti-Iba1 (1/500, Wako, ref. W1W019-19741), polyclonal rabbit anti-fibronectin (1/250, Abcam, ref. ab2413), and polyclonal goat anti-choline acetyltransferase (1/200, Merck Millipore, ref. AB144P). Explant wholemounts or cryosections were washed in DPBS and incubated with an Alexa Fluor 594-conjugated donkey anti-mouse immunoglobulin (1/500, Thermo Fisher Scientific, ref. A21203), an Alexa Fluor 488-conjugated donkey anti-rabbit immunoglobulin (1/500, Thermo Fisher Scientific, ref. A21206), or an Alexa Fluor 594-conjugated donkey anti-goat immunoglobulin (1/500, Thermo Fisher Scientific, ref. A11058) as secondary antibodies (Thermo Fisher Scientific), and the nuclei were stained with Dapi (1/500) for 1 h, at RT. Then, retinal explants, wholemounts or cryosections, were washed and mounted in Fluoromount (Sigma Aldrich, ref. F4680-25ML).

### Histopathological analysis of retinal explants

Retinal explant cryosections 12 μm in thickness were stained with hematoxylin and eosin (H&E). Stained cryosections were scanned at ×100 and ×200 magnifications with the digital whole-slide scanner NanoZoomer 2.0 HT scanner (Hamamatsu Photonics), using the NanoZoomer’s 3-CCD TDI camera. NDP viewer software (Hamamatsu Photonics) was used to measure the thickness of the retinal explants and analyze their histology.

### Immunohistological quantification

For RGC quantification in wholemount retinas, imaging was performed at ×200 magnification (306 µm^2^) using an epifluorescence microscope (Zeiss AX-10). RGCs were identified based on Brn3a and RBPMS markers. Images were taken at four locations from the optic nerve to the peripheral retina for each explant. Cell counts were performed manually using image analysis software (ImageJ, United States National Institutes of Health (NIH)) and *cell counter plugin* and expressed as RGC/306 µm^2^ or percentage of cells compared to Days Ex Vivo (DEV) 0. Concerning quantification in transverse sections, sections were taken throughout the whole explant, with approximately 18 slides/explants, allowing placement of different areas of the explant on the same slide. Three images were acquired per section, in five different sections on a slide. RGC or displaced amacrine cells (DAC) quantification was expressed as RGC/mm or DAC/mm. For GFAP, Iba1 and CD68 immunostaining quantification, explant cryosections were taken using an epifluorescence microscope (Zeiss AX-10). Three images were acquired for each explant using a ×10 or ×20 objective and the same parameters of time exposure for each explant. NIH Image J software was then used to quantify the Raw Integrated Density of each section, allowing us to obtain the sum of pixels values. For all images, Background values have been subtracted of Raw Integrated Density and the same area was selected. Sections were analyzed in a blinded manner; the experimenter was blinded to the treatment.

### Quantitative RT-PCR

Total RNA of 6–16 retinal explants per group (3 mg/explant) was purified using the NucleoSpin RNA XS kit (Macherey–Nagel, ref. 740902.50) according to protocol. Total RNA was reverse-transcribed into cDNA a High Capacity RNA to cDNA kit (Life Technologies, ref. 4368814) according to the manufacturer’s instructions. Real-time PCR was performed using the TaqMan Gene Expression PCR Master mix (Applied Biosystems, ref. 4324020) and the Applied Biosystem Fast 7500 (Applied Biosystems). The delta–delta *Ct* method (dd*Ct*) was used to analyze the relative gene expression; that is, the fold change of mRNA upon downregulation of various target genes was determined. Rps18 was used as a housekeeping gene.

### Statistical analysis

Statistical analyses were performed using GraphPad Prism 7. Data are presented as mean ± SEM. Student’s *t*-test or Mann–Whitney was performed for unpaired comparisons between RGC viabilities after counting with Brn3a and RBPMS staining and relative mRNA levels. Unpaired* t*-test was used to quantify neurons after exposure to pharmacological agents (BDNF and NMDA), and two-way ANOVA or Mann–Whitney was used for retinal explant cocultures with MSCs. RT-qPCR data were analyzed using one-way ANOVA, Student’s *t*-test, or Mann–Whitney for unpaired comparisons. One-way ANOVA was used to quantify GFAP, Iba1 and CD68 immunostaining.

## Results

### Isolation and characterization of rat bone marrow mesenchymal stem cells

Rat BMMSCs from three different production batches were characterized using FCM analysis. The data showed that MSCs highly expressed positive MSC markers CD29, CD73, and CD90 and did not express CD11b/c, CD45, CD68, and the MHC Class II (Ia) antigen in accordance with the International Society for Cellular Therapy [32]. Overlaid fluorescence histograms of specific markers (in black line *vs* negative control in grey), percentages of positive cells and mean fluorescence intensities are presented in the Additional file 1.

### RGC degeneration in a retinal explant culture model

**Fig. 1:**
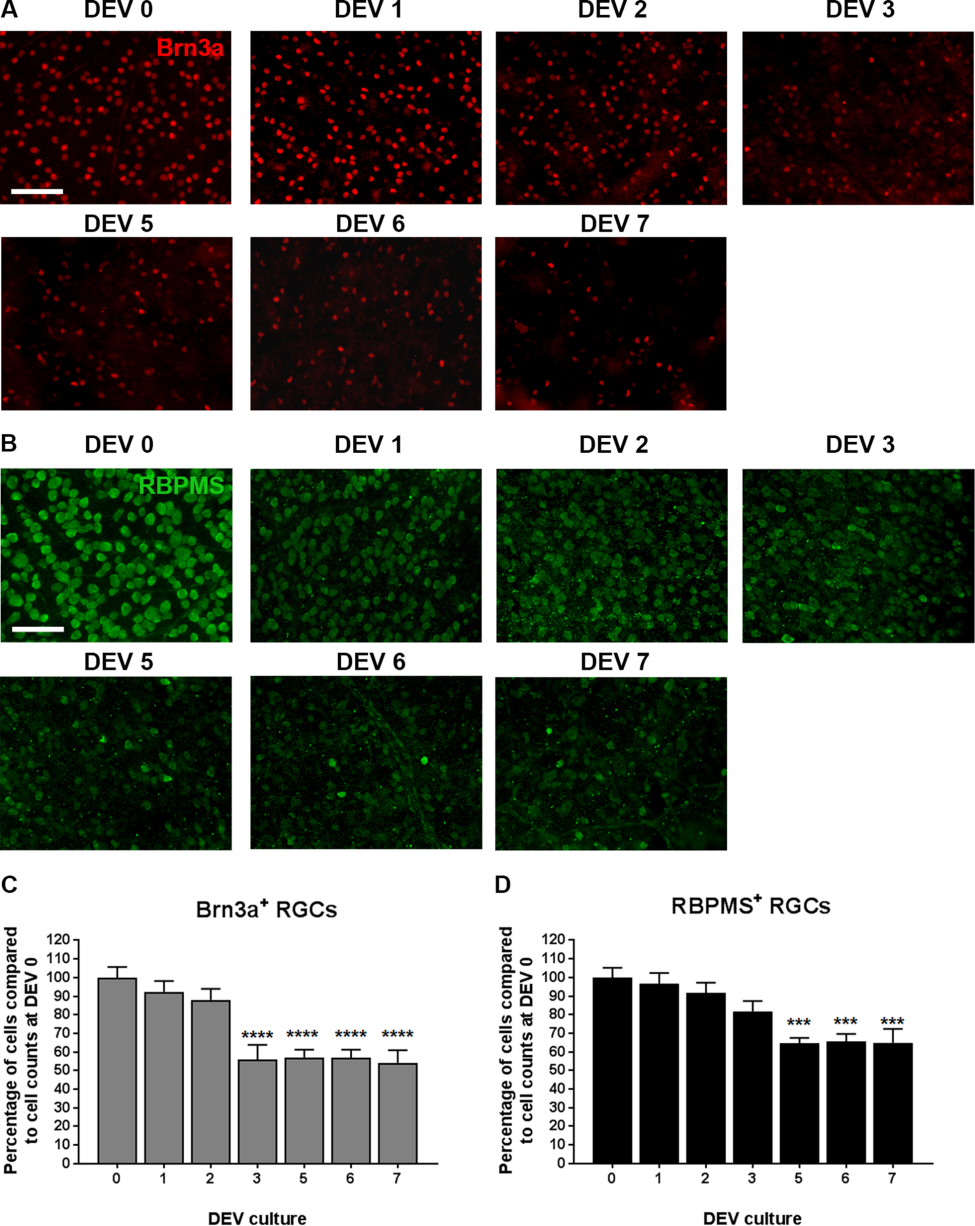
Representative images of wholemount retinal explants (*n* = 8 per day) in culture from DEV0 to DEV7 immunolabeled with Brn3a (**A**) and RBPMS (**B**) at ×200 magnification (scale bar = 100 µm). Quantification of Brn3a+ RGCs (**C**) and RBPMS+ RGCs (**D**) from wholemount retinal explants was expressed as percentage of DEV 0 (defined as 100%). Error bars are standard error of the mean. ***P < 0.001, ****P < 0.0001

The best therapeutic window to assess neuroprotection was determined by evaluation of RGCs degeneration following optic nerve axotomy in the retinal explant culture model, which can vary between laboratories, handlers, or culture conditions. Brn3a and RBPMS specific RGCs markers were used for RGCs counting from ‘Day ex vivo’ (DEV) 0–7 (Fig. 1A–D) [34, 35]. Loss of RGCs occurred with both markers after 24 h of culture but without significance. The number of RGCs was statistically different at DEV 3 and later compared to DEV 0 for Brn3a quantification (44% RGCs death at DEV 3 from DEV 0, *P* < 0.0001) or DEV 5 for RBPMS quantification (36% RGCs death compared DEV 0, *P* < 0.0001) (Fig. 1). From DEV 5 to DEV 7, a plateau of the RGCs loss curves for Brn3a and RBPMS quantification was obtained. From these results, we determined an optimal therapeutic window from DEV 5 to 7 of culture for testing neuroprotective agents or MSCs.

### Retinal explant responses to neuroprotective or excitotoxic stimuli

**Fig. 2:**
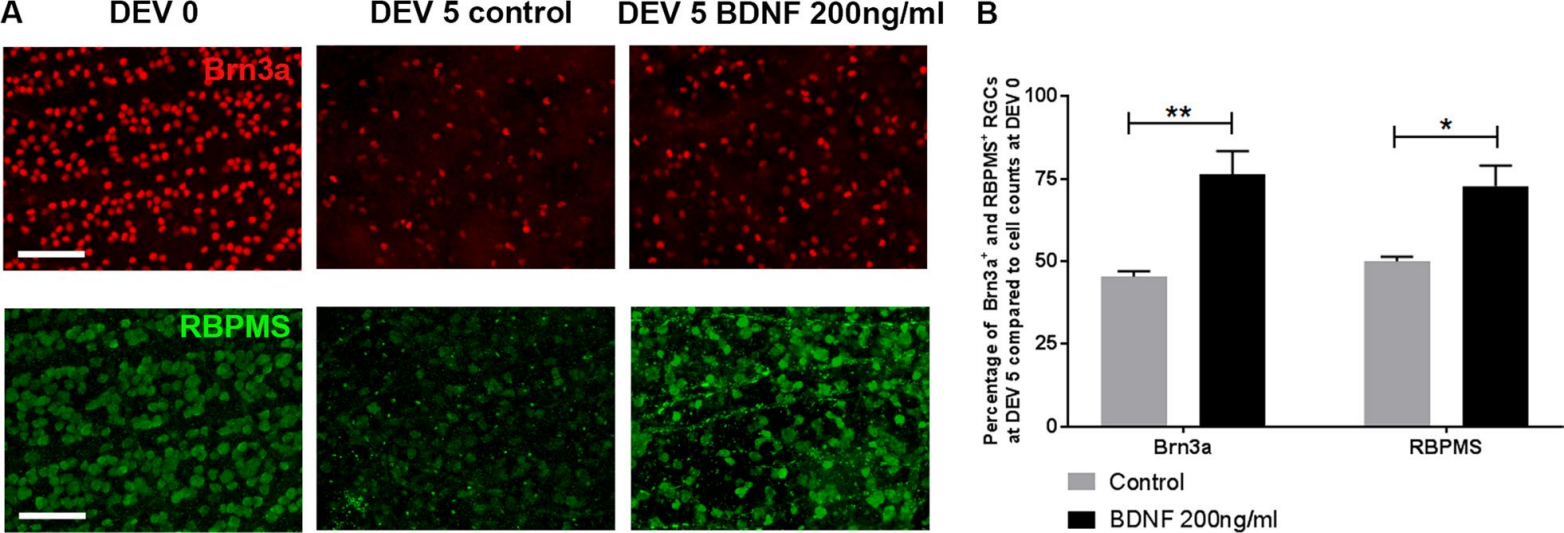
**A** Representative images of wholemount retinal explants (*n* = 4/day) in culture at DEV 0 and DEV 5, immunolabeled with Brn3a (red) and RBPMS (green) at ×200 magnification (scale bar = 100 µm) treated daily with BNDF (200 ng/ml). **B** Quantification of Brn3a+ or RBPMS+ RGCs in control group (grey bar) and Brn3a+ or RBPMS+ RGCs in BDNF-treated group (black bar) at DEV 5 from wholemount retinal explants was expressed as percentage of DEV 0 (defined as 100%). Error bars are standard error of the mean. *P < 0.05, **P < 0.01

To evaluate the ability of the retinal explant model to respond to neuroprotective agents, we exposed explants for 5 days to BDNF [36, 37]. At DEV 5 with Brn3a and RBPMS markers, quantification of RGCs on wholemount retinas confirmed the neuroprotective effect of BDNF, with a significant increase in the percentage of Brn3a+ RGCs survival at DEV 5 in treated group compared to control group (76.38% ± 7.08% vs. 45.47% ± 1.55%, *P* = 0.0053) and RBPMS+ RGCs survival at DEV 5 in treated group compared to control group (72.81% ± 6.16% vs. 50.06% ± 1.37%, *P* = 0.0113) (Fig. 2). Retinal explants significantly respond to a neuroprotective stimulus.

**Fig. 3:**
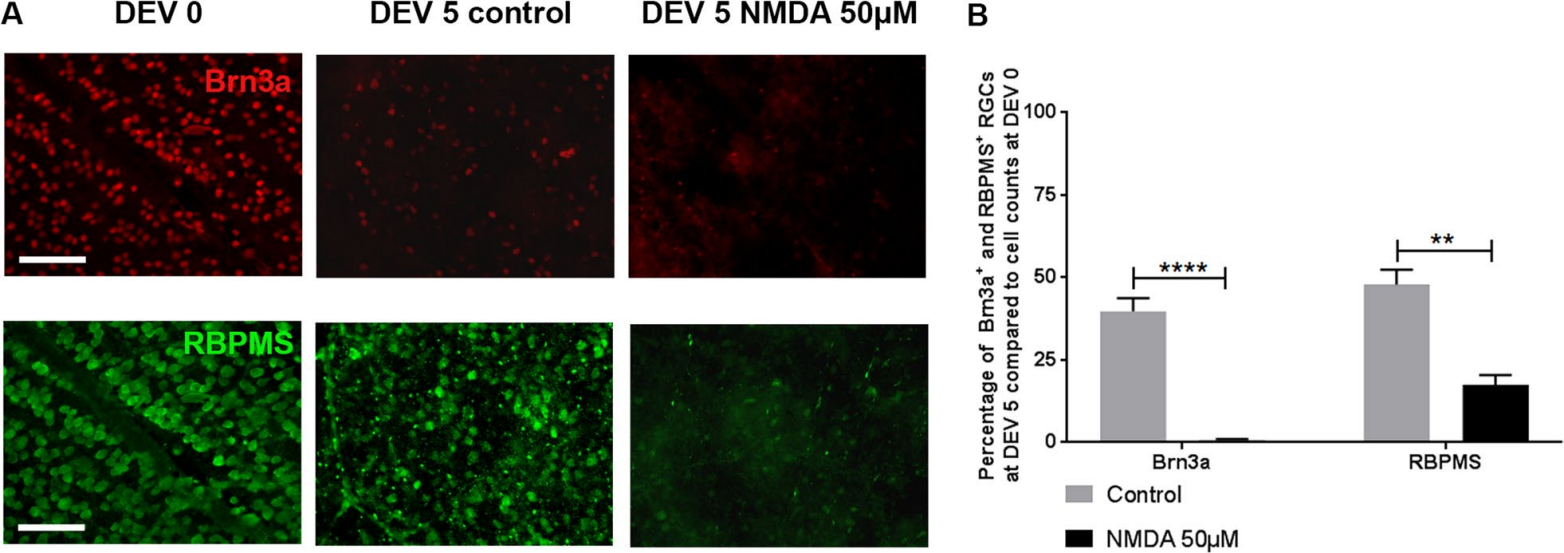
**A** Representative images of wholemount retinal explants (*n* = 4/day) in culture at DEV 0 and DEV 5, immunolabeled with Brn3a (red) and RBPMS (green) at ×200 magnification (scale bar = 100 µm) treated daily with NMDA (50 µM). **B** Quantification of Brn3a+ or RBPMS+ RGCs in control group (grey bar) and Brn3a+ or RBPMS+ RGCs in NMDA-treated group (black bar) at DEV 5 from wholemount retinal explants was expressed as percentage of DEV 0 (defined as 100%). Error bars are standard error of the mean. Error bars are standard error of the mean. **P < 0.01, ****P < 0.0001

To evaluate the ability of the retinal explant model to respond to excitotoxic agents, we exposed explants for 5 days to NMDA [36, 37]. At DEV 5 NMDA exposure at 50 µM caused significant decrease in RGCs survival compared to controls with both markers Brn3a (0.65% ± 0.33% vs. 39.73% ± 3.98%, *P* < 0.0001) and RBPMS (17.46% ± 2.91% vs. 47.75% ± 4.53%, *P* = 0.0014) (Fig. 3). Retinal explants significantly respond to an excitotoxic stimulus.

### Coculture of MSCs with retinal explants confers RGC neuroprotection

**Fig. 4:**
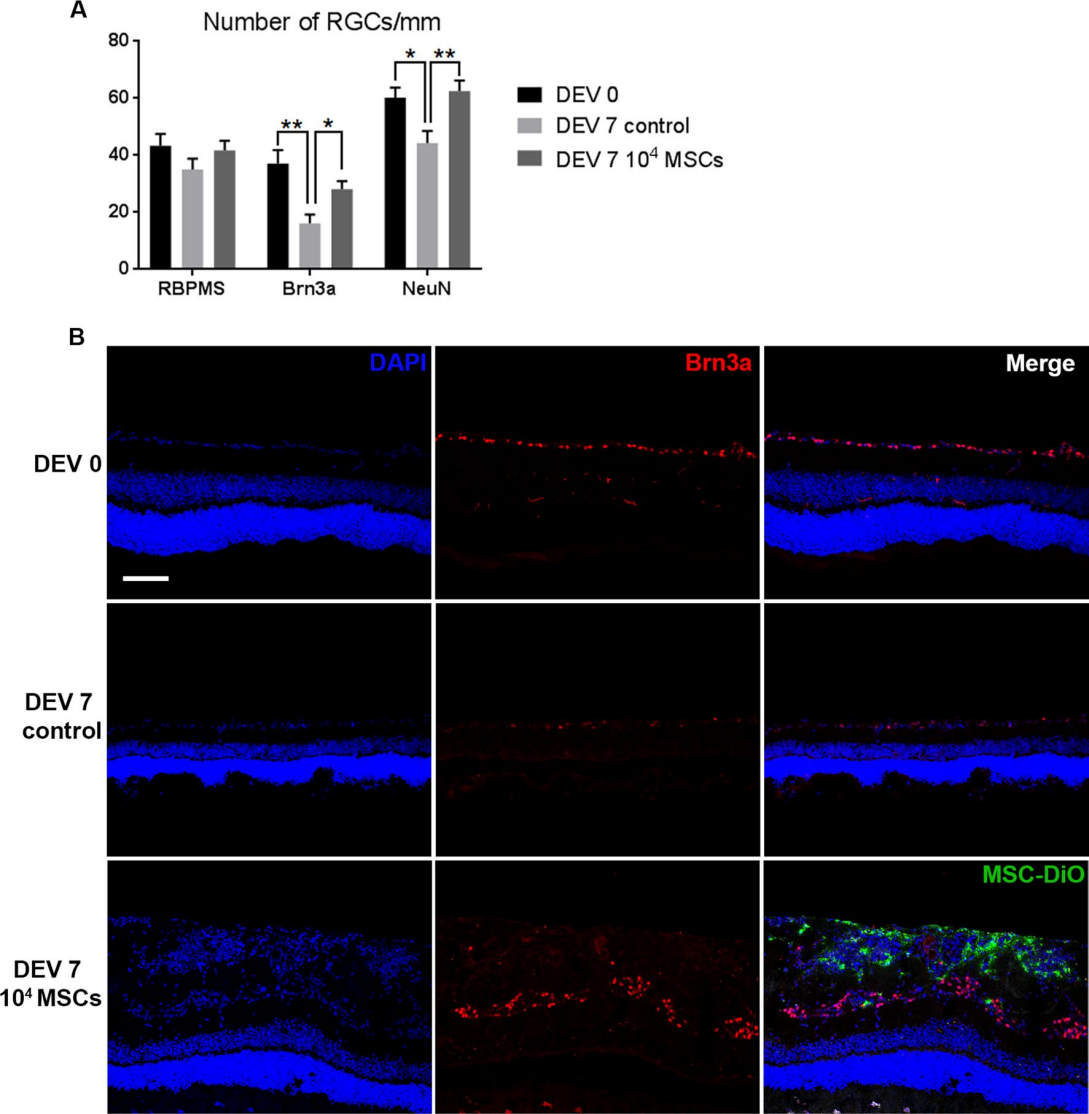
**A** Quantification of RBPMS+, NeuN+ and Brn3a+ RGCs from retinal explants (*n* = 6/day) cocultured with 1.10^4^ MSCs for 7 days. RGC counts on cryosections are expressed as RGCs/mm. *P < 0.05, **P < 0.001. **B** Representative images of retinal explant cryosections cocultured with 1.10^4^ MSCs for 7 days at DEV 0 and DEV 7, immunolabeled with Brn3a (red) and DiO-labeled MSCs (green) at ×200 magnification (scale bar = 100 µm)

In accordance with the publications of Johnson et al., we investigated whether the presence of MSCs could limit or prevent RGC loss in retinal explants after an even longer duration, namely at DEV 7 [33, 38]. At DEV 7, the number of RGCs stained with Brn3a and NeuN was significantly higher in cryosections of retinal explants cocultured with 1.10^4^ MSCs compared to the control group (28.13 ± 4.61/mm vs 16.16 ± 5.85/mm, *P* = 0.0490 and 62.55 ± 6.92/mm vs 44.39 ± 8.26/mm, *P* = 0.0018, respectively). RBPMS quantification did not show statistically significant differences at DEV 7 between control and MSC coculture groups. No significant difference in RGC numbers was found between DEV 0 and DEV 7 with 10^4^ MSCs for RBPMS, Brn3a, or NeuN staining (Fig. 4). However, NeuN being a neuronal marker expressed by both RGCs and DACs, we specifically quantified DACs in RGC layer using an anti-choline acetyltransferase (ChAT) antibody in order to avoid a bias in RGCs survival counting using this marker. We found no significant difference in ChAT+ DACs between group at DEV 0 and DEV 7 (Additional file 2). Thus, RGCs survival estimated with NeuN is not influenced by potential NeuN+ DACs in the RGC layer. MSCs reduced RGC loss in retinal explants at DEV 7.

### MSCs decrease gliosis in retinal explants

**Fig. 5:**
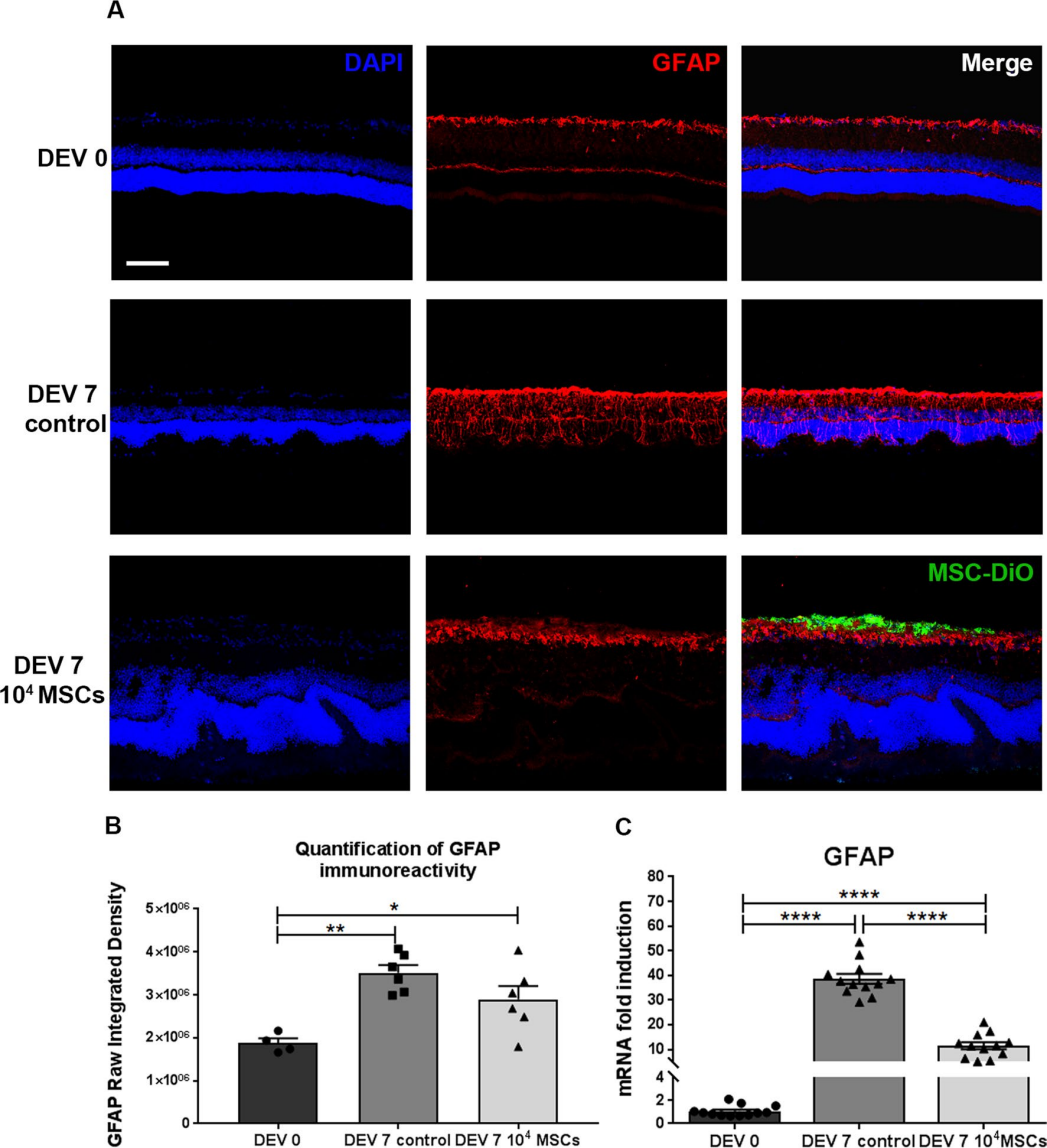
**A** Representative images of retinal explant cryosections cocultured with 1.10^4^ MSCs for 7 days at DEV 0 and DEV 7, immunolabeled with GFAP (red) and DiO-labeled MSCs (green) at ×200 magnification (scale bar = 100 µm). **B** Quantification of GFAP immunoreactivity (expressed as Raw Integrated Density). *n* = 4–6 animals/group. Ordinary one-way ANOVA was performed. *P < 0.05, **P < 0.01. **C** RT-qPCR analysis of GFAP expression in retinal explants at DEV 0 and DEV 7. mRNA levels are presented after normalization with the housekeeping gene Rps18. *n* = 12 animals/group. Unpaired *t*-test was performed for unpaired comparisons. ****P < 0.0001

To determine the inflammatory response following MSCs implantation, we analyzed GFAP immunostaining and GFAP mRNA expression in retinal explants at DEV 0 and DEV 7. GFAP immunoreactivity in astrocytes and Müller cells was upregulated in all retinal layers at DEV 7 in the control group compared to DEV 0, where GFAP was limited to the nerve fiber layer (NFL) and outer plexiform layer (OPL). In contrast, at DEV 7 in the MSC coculture group, GFAP immunostaining was limited to the NFL and to a lesser extent to the OPL (Fig. 5A). Moreover, we measured the Raw Integrated Density at DEV 0 and DEV 7 in the control group and the MSCs coculture group. This semi-quantitative analysis clearly demonstrated the significant upregulation of GFAP staining at DEV 7 in both groups confirming the glial activation (*P* = 0.0015 and *P* = 0.0361, respectively) compared to DEV 0 (Fig. 5B). Nonetheless, no significant difference was observed between the control and the MSC coculture group at DEV 7. GFAP mRNA was significantly higher at DEV 7 in the control and MSC coculture groups (38.5-fold, *P* < 0.0001 and 11.4-fold, *P* < 0.0001, respectively) compared to DEV 0. However, GFAP mRNA was significantly lower at DEV 7 in the MSC coculture group compared to the control group (*P* < 0.0001) (Fig. 5C). These data demonstrate that MSC coculture conferred limited glial activation in retinal explants at DEV 7 compared to the control group. However, GFAP activation was limited but still robust in the NFL and OPL layer in the MSC coculture group, proving a significant, localized reactive gliosis following MSC implantation.

### MSCs reduce microglial activation

**Fig. 6:**
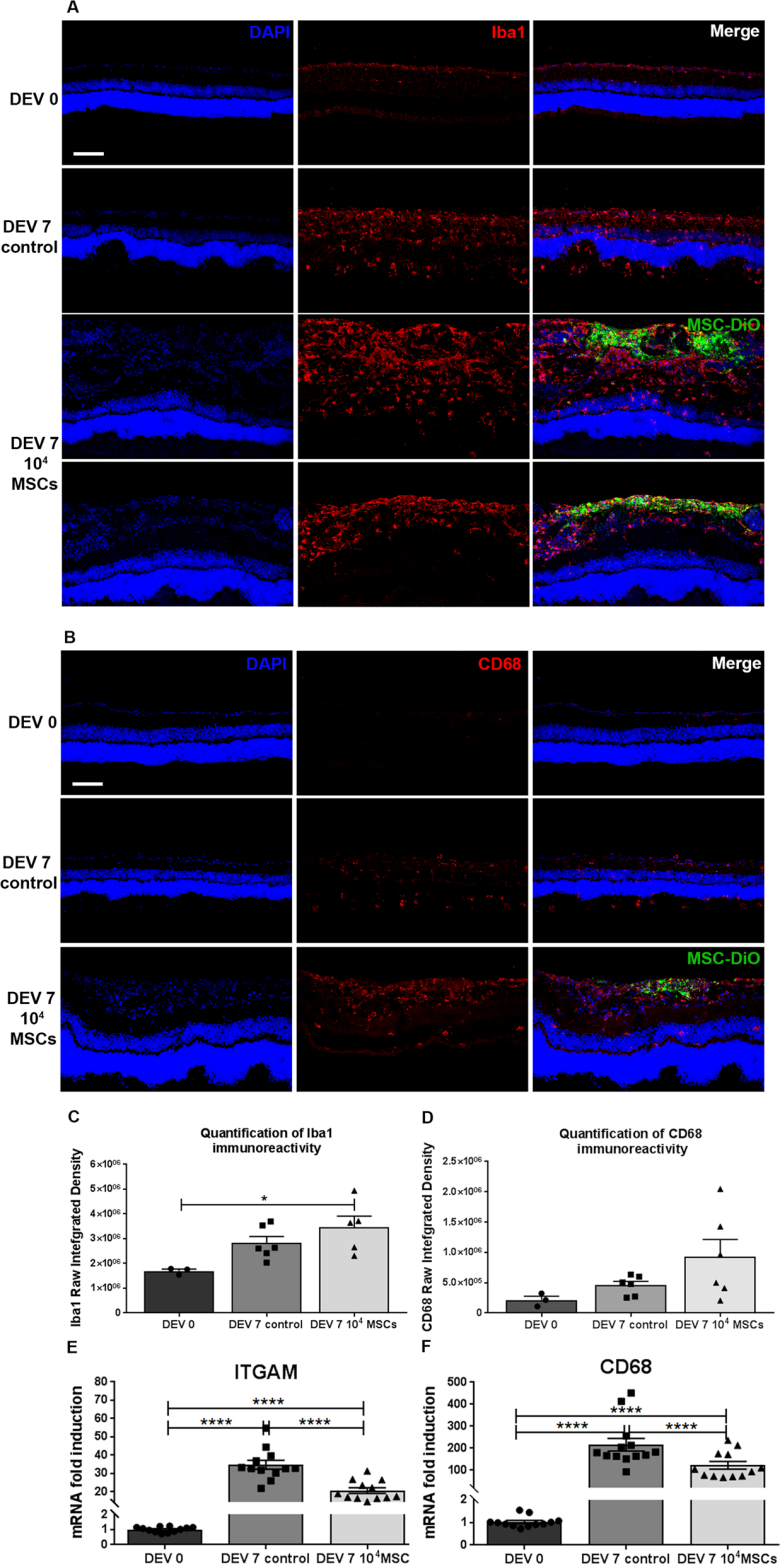
Representative images of retinal explant cryosections cocultured with 1.10^4^ MSCs at DEV 0 and DEV 7 immunolabeled with Iba1 (red) (**A**), CD68 (red) (**B**) and DiO-labeled MSCs (green) at ×200 magnification (scale bar = 100 µm). **C** Quantification of Iba1 immunoreactivity (expressed as Raw Integrated Density). *n* = 4–6 animals/group. Ordinary one-way ANOVA was performed. *P < 0.05. **D** Quantification of CD68 immunoreactivity (expressed as Raw Integrated Density). *n* = 4–6 animals/group. Ordinary one-way ANOVA was performed. **E**, **F** RT-qPCR analysis of ITGAM and CD68 expression in retinal explants at DEV 0 and DEV 7. mRNA levels are presented after normalization with the housekeeping gene Rps18. *n* = 12–13 animals/group. Unpaired *t*-test was performed for unpaired comparisons. ****P < 0.0001

To determine the microglial cell response following coculture with MSCs, we analyzed ionized calcium binding adaptor molecule 1 (Iba1) and CD68 immunostaining and ITGAM and CD68 mRNA expressions in retinal explants at DEV 0 and DEV 7. At DEV 0, Iba1+ microglial cells were located in the inner plexiform layer (IPL) and ganglion cell layer (GCL), and no CD68+ cells were found in retinal layers. At DEV 7 in the control group, Iba1+ and CD68+ cells were found in all retinal layers, contrary to the MSC group, where Iba1+ and CD68+ cells were limited to the inner retinal layers (GCL, IPL and inner nuclear layer (INL)). Moreover, at DEV 7 in the MSC group, microglial cells were present mainly at the interface between the GCL and MSCs (Fig. 6A, B). Then, we quantified Iba1 and CD68 Raw Integrated Density at DEV 0 and DEV 7 in the control and MSC coculture groups. It demonstrates the significant upregulation of Iba1 staining at DEV 7 in MSC coculture group compared to DEV 0 (*P* = 0.0235). Moreover, no significant difference was observed between control group at DEV 7 and MSC coculture or DEV 0 groups (Fig. 6C). Concerning CD68 staining, no significant difference was observed in all groups, despite a slight tendency to fluorescence increase at DEV 7 for both groups (Fig. 6D). RT-qPCR analysis showed that ITGAM and CD68 mRNA fold inductions were significantly lower in the MSC group compared to the control group (respectively, 17.3 vs. 33.1 and 83.4 vs. 169, *P* < 0.0001) (Fig. 6E, F). These data demonstrate that microglial cells were distributed differently throughout the retina at DEV 7 in the control and MSC groups, with a limited but strong distribution of microglial cells to the internal retinal layers in the MSC group. In the control group at DEV 7, Iba1+ microglial cells migrated and proliferated toward the outers layers of the retina. Furthermore, microglial activation and proliferation were higher at DEV 7 in the control group compared to the MSC coculture group. However, in the MSC coculture group, this microglial activation was concentrated at the RGC/MSC interface. MSC limited the microglial activation in retinal explants.

### MSCs have an immunomodulatory effect on retinal explants

**Fig. 7:**
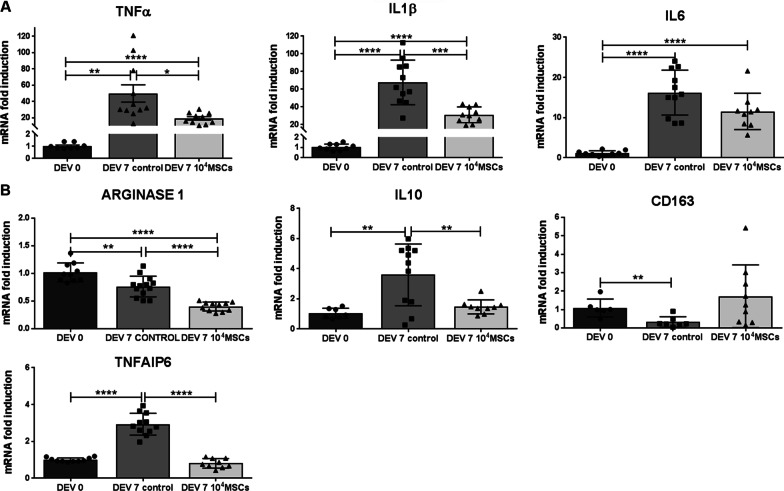
RT-qPCR analysis of M1 (**A**) and M2 (**B**) phenotype marker expression in retinal explants at DEV 0 and DEV 7. mRNA levels are presented after normalization with the housekeeping gene Rps18. *n* = 6–16 explants/group. Unpaired *t*-test or Mann–Whitney test were performed for unpaired or nonparametric comparisons. *P < 0.05, **P < 0.01, ***P < 0.001, ****P < 0.0001

To investigate whether the presence of MSCs in the retinal explant coculture model could exert immunomodulatory properties and influence on microglial phenotypes, we analyzed microglial polarization markers through the mRNA expression of type M1 *classically activated*, namely TNFα, IL1β and IL6, and M2 *alternatively activated*, namely Arginase 1, IL10, CD163 and TNFAIP6 [15, 20]. Our data showed that mRNA expression of M1 phenotype markers TNFα, and IL1β levels were significantly lower at DEV 7 in the MSC group compared to the control group (*P* = 0.0143 and *P* < 0.001, respectively) (Fig. 7A). There was a significant increase in the level of IL6 mRNA expression at DEV 7 but no significant difference between the control group and the MSC coculture group. The mRNA expression levels of the markers of M2-polarized microglia, Arginase 1, IL10, and TNFAIP6 were significantly lower in the MSCs coculture group at DEV 7 compared to the control group (*P* < 0.0001, *P* = 0.071, and *P* < 0.0001, respectively) (Fig. 7B). Only mRNA expression of the M2 marker CD163 was significantly lower in the control group compared to DEV 0, with no significant difference between the MSC coculture group and the control group at DEV 7. However, there were no significant difference between the DEV 7 MSC group and DEV 0 for IL10, CD163 or TNFAIP6 mRNA expression levels. MSCs reduced the expression of M1 inflammatory markers.

### MSCs affect retinal architecture and induce an ERM-like phenotype

Before the coculture, DiO labeling of MSCs allowed tracking of the MSCs to determine their ability to graft into the retinal explant. At DEV 7, a few MSCs were found in the retinal explants at the GCL level. Most MSCs did not penetrate the retina and remained at the surface of the explant, probably where the 2 µl of MSC suspension was deposited (Fig. 3B).

**Fig. 8:**
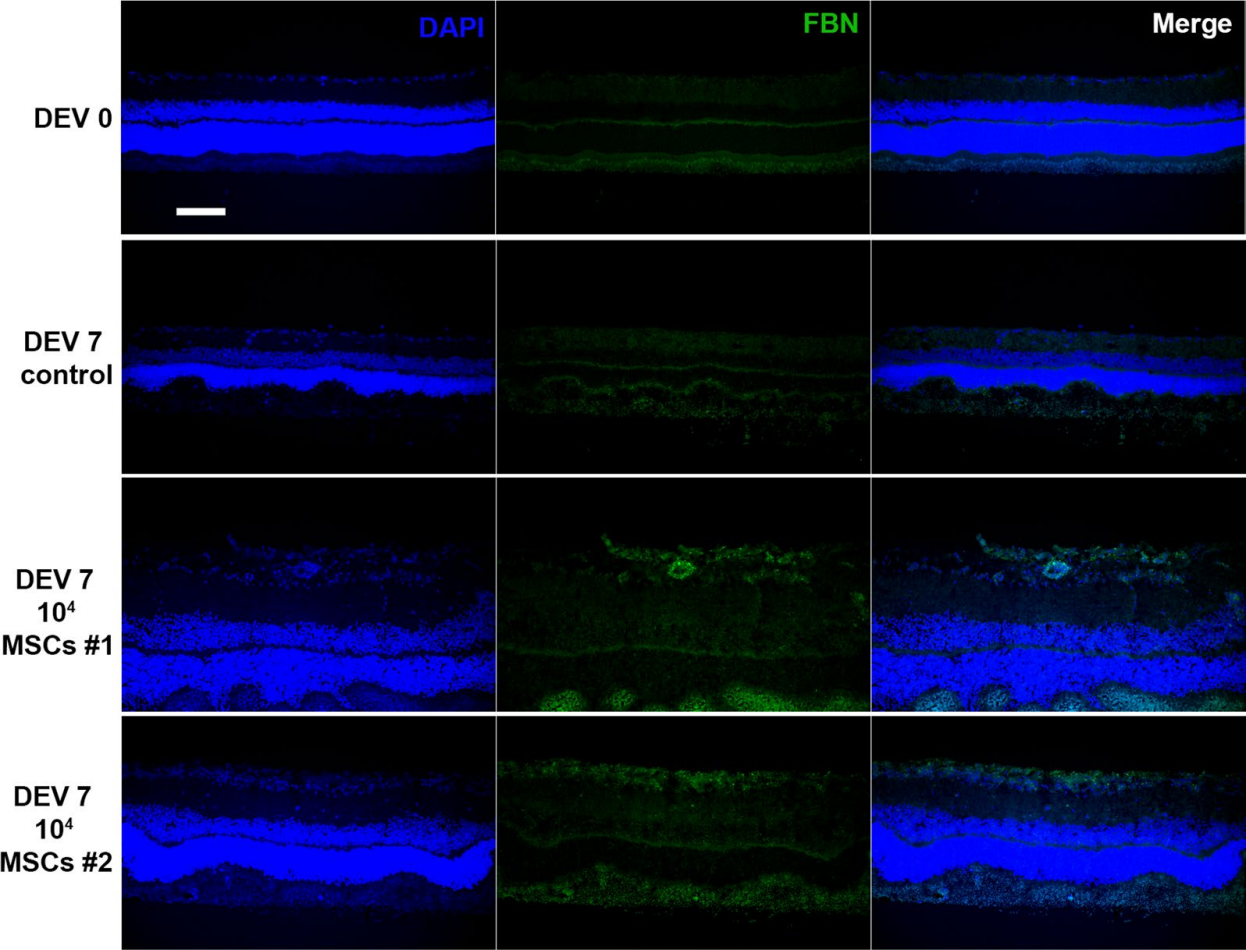
Representative cryosections of retinal explants cocultured with 10^4^ MSCs at DEV 0 and DEV 7, immunolabeled with fibronectin (green) at ×200 magnification (scale bar = 100 µm) (*n* = 3–5/group)

**Fig. 9:**
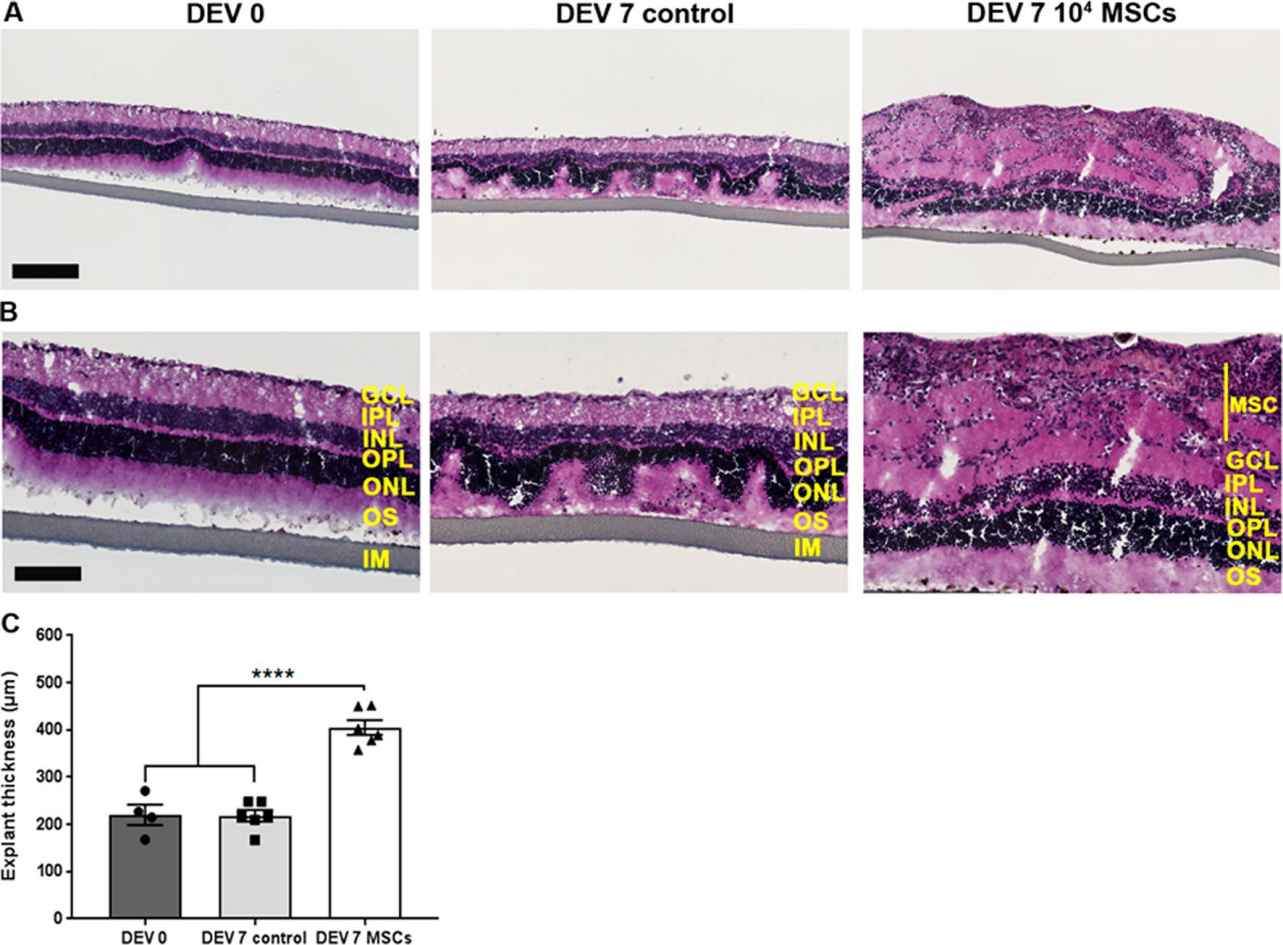
**A** Representative hematoxylin and eosin (H&E) staining of retinal explant cryosections cocultured with 10^4^ MSCs at Days Ex Vivo 0 and Ex Vivo 7 at ×100 magnification (scale bar = 200 µm) and **B** at ×200 magnification (scale bar = 100 µm). *MSC* mesenchymal stem cell, *GCL* ganglion cell layer, *IPL* inner plexiform layer, *INL* inner nuclear layer, *OPL* outer plexiform layer, *ONL* outer nuclear layer, *OS* outer segments, *IM* insert membrane. **C** Retinal explant thickness measurement. Each bar is the mean ± SEM. *n* = 4–6/group. Ordinary one-way ANOVA was performed. ****P < 0.0001

Explant “swelling” was observed in all explants in the MSC cocultured group, with increased explant thickness and retinal folding, compared to DEV 0 and to DEV 7 controls. MSCs also induced the appearance of an epiretinal membrane at the surface of the retinal explants. In order to investigate whether this distortion was associated with an epiretinal membrane phenotype, we used an anti-fibronectin antibody, since this protein is known to be upregulated in idiopathic epiretinal membranes [39]. Figure 8 shows the increase in fibronectin labelling at the surface of the explant cocultured with MSCs. This fibronectin expression was higher on the apical side of the explant, at the contact area between the MSCs and GCL. Likewise, H&E staining was performed to assess retinal micro-architectural organization and to quantify explant swelling in presence of MSCs. The laminar structure of the retinal explants at DEV 7 in the control group showed a folded appearance to the outer segments (OS) and outer nuclear layer (ONL) compared to DEV 0 (Fig. 9). Explants in the coculture group exhibited a thicker internal retinal layer. Figure 9C shows a significant increase in retinal explant thickness, which was found in all the explants in the MSCs cocultured group compared to the DEV 7 control and DEV 0 groups (405 ± 30 µm vs 218 ± 20 µm and 220 ± 28 µm, respectively, *P* < 0.0001). MSCs induced thickening of the explants and disorganization of the retinal architecture with a formation of an epiretinal membrane.

## Discussion

Over the last decade, there has been increasing interest in the use of stem cells, including retinal progenitor cells (RPCs), embryonic stem cells (ESCs), induced pluripotent stem cells (iPSCs) and MSCs to regenerate RGCs in glaucoma [40]. MSCs have also shown neuroprotective and immunomodulatory properties. They have the advantages of demonstrating immunosuppressive effects and are less immunogenic and tumorigenic than ESCs. Compared with harvesting RPCs, it is relatively easy to obtain MSCs, and they possess a higher proliferative capacity [2]. The iPSCs strategy is also interesting, as these cells have the potential for reducing immunogenicity through autologous transplantation, but iPSCs have a lower variable differentiation efficiency and a relatively high risk of gene mutation [41].

Despite promising results in animals, clinical trials of intravitreal injections of BMMSCs have raised some safety concerns, which require further studies and the development of additional experimental models mimicking retinal degeneration in glaucoma [2].

In the present study, we showed that our ex vivo axotomy model results in rapid RGC degeneration and gliosis caused by optic nerve transection and disruption of axonal transport, enabling investigation of neuroprotective or anti-inflammatory therapeutic compounds or stem cell transplantation therapies in human or rodent tissues [42, 43]. This retinal explant model has the advantage of maintaining an in vivo type architecture with all the neuroretina layers retaining intercellular interactions. It also limits the number of animals killed, since one animal provides eight explants. Thus, this model fills the gap between relevant but time/cost/animal-consuming preclinical models and cell culture models which cannot substitute for the complexity of an entire tissue.

Advances in glaucoma research increasingly suggest that this degenerative optic nerve disease is linked to neuroinflammation [10]. The activation of microglia seems to play an important role in glaucoma pathogenesis, and strategies aiming to modulate reactive microglia are explored to slow down the progression of glaucoma and improve RGC survival [9]. In this study, we show that the MSCs have immunomodulatory properties and could be able to block RGC death in the retinal explant axotomy model by modifying the inflammatory state.

Although MSCs allowed us to observe neuroprotection, reduced gliosis, and modulation of inflammation in our ex vivo retinal explant model, we noticed edema and folding of the retinal explants.

This swelling of the explants may correspond to the adverse effects reported in clinical trials in humans. Indeed, despite promising results in animals with good efficacy and good safety profiles, the translation to humans in clinical trials was more than disappointing [25]. Several studies and trials have warned of serious ocular adverse effects following MSC transplantation, questioning the safety of using MSCs in retinal disease [44–46]. Among the complications reported, retinal detachments, retinal folds, subretinal exudative fluid, vitreous hemorrhage, vitreoretinal proliferation, proinflammatory vitreous clumping, thick epiretinal membrane formation as well as ocular hypertension and microcystic corneal edema have been described [45, 47–49]. Surprisingly, these effects were not found in a phase 1 trial studying the safety of intravitreal autologous MSC transplantation in 14 patients with retinitis pigmentosa [50]. This could be due to the atrophic status of the retina in such severe degenerations. In a clinical phase I study, Satarian et al. described severe fibrous tissue proliferation in the BMMSC-injected eye of one patient, which was reproduced in a mouse vitreous cavity injected with the same MSCs [49]. MSCs of the other two patients did not generate fibrosis in the animal vitreous. Considering the heterogeneity of individual MSC samples, they thus proposed evaluation of the cells in animals prior to their intravitreal injection in patients.

Possible explanations for the disorganization found in our explants following coculture with MSCs could include differentiation of MSCs into myofibroblasts, promoting retinal fibrosis [51]. We noticed an increase of gliosis and microglial activation at the junction between the explants and MSCs. Tassoni et al. also demonstrated in both in vivo and ex vivo mice retina that intravitreal BMMSC transplantation was associated with gliosis-mediated retinal folding, upregulation of intermediate filaments, and recruitment of macrophages. They described a JAK/STAT3 and MAPK (ERK1/2 and JNK) cascade activation in retinal Müller glia [52].

The question, therefore, arises of finding a method allowing preservation of the beneficial effects of MSCs while avoiding the undesirable effects and disadvantages of using MSCs, such as potential tumorigenicity, need for autologous collection, and variability.

It was initially hypothesized that cell replacement was an important mechanism of action of MSCs. However, considering the poor ability to graft MSCs into the retina [4, 33], the beneficial effects of MSCs are now believed to be mediated mostly by their paracrine ability to release multiple factors such as neurotrophins, growth factors (BDNF, NGF, PDGF), chemokines, immunomodulators (IDO, PGE2, TSG-6) and extracellular vesicles [26, 53, 54]. This broad range of released factors with diverse functions, including anti-inflammatory potential, neuroprotection, and immunomodulation, is known as the secretome or conditioned medium (CM). Concerning the immunomodulatory effects of MSCs, we were able to show in our model that MSCs reduced the expression of M1 state as well as M2 state cytokine mRNAs. Therefore, MSCs induced an immunosuppressant effect, but failed to promote M2 subtype polarization. Such properties of MSCs have been found in several studies [20, 55]. Holan et al. demonstrated that the cytokine environment of MSCs influences the spectrum of cytokines they produce, and thus, their immunoregulatory potential [56]. To enhance the efficiency of such CM, several priming methods, such as culture duration, O_2_ level, addition of growth factors or anti/pro-inflammatory cytokines, or three-dimensional culture methods, could allow switching the factors produced by MSCs towards an anti-inflammatory profile [57–60].

More recently, other stem cell-free approaches using extracellular vesicles (exosomes and microvesicles), have been under investigation. These vesicles are considered to be responsible for the paracrine effects of MSCs, promoting immunomodulation, tissue repair, and regeneration, with a lower risk of oncogenic transformation and immune reactions than injections of whole MSCs [25, 61–63].

## Conclusion

Using an ex vivo retinal explant model, we demonstrated a neuroprotective and immunomodulatory effect of MSCs on RGCs. Since this model allowed us to reproduce the expected but also undesirable effects of injections of MSCs, it appears suitable for screening efficacy and safety of potential candidates for retinal therapy and should be useful for future assessment of cell-based or alternative methods of neuroprotection and neuroregeneration.

## Supplementary Information

**Additional file 1.** Flow cytometric images showing the histogram overlays of specific antibodies vs isotypic-matched immunoglobulins for each marker from one BMMSC production batch with table presenting the percentages of positive cells and means of fluorescence intensities (MFI) for each marker and each production, with their means, and standard error of the means (SEM).

**Additional file 2.** **A** Representative images of retinal explant cryosections cocultured with 1.10^4^ MSCs for 7 days at DEV 0 and DEV 7, immunolabeled with ChAT (red) and DiO labeled MSCs (green) at ×200 magnification (scale bar = 100 µm). **B** Quantification of ChAT+ DACs from retinal explants (*n* = 4–6/day) co-cultured with 1.10^4^ MSCs for 7 days. DACs counts on cryosections are expressed as DACs/mm.

## Data Availability

The datasets used and/or analyzed during the current study are included in this published article or available from the corresponding author on reasonable request.

## Abbreviations

ARG1: Arginase 1
BDNF: Brain-derived neurotrophic factor
BMMSC: Bone marrow mesenchymal stem cell
ChAT: Choline acetyl transferase
CM: Conditioned medium
DAC: Displaced amacrine cells
DEV: Days ex vivo
ESC: Embryonic stem cells
FC: Flow cytometry
GCL: Ganglion cell layer
H&E: Hematoxylin and eosin
INL: Inner nuclear layer
IPL: Inner plexiform layer
iPSC: Induced pluripotent stem cells
MSC: Mesenchymal stem cell
NMDA: *N*-methyl-d-aspartate
ONL: Outer nuclear layer
OPL: Outer plexiform layer
OS: Outer segments
PTFE: Polytetrafluoroethylene
RGC: Retinal ganglion cell
ROS: Reactive oxygen species
RPC: Retinal progenitor cells
RT: Room temperature
TSG-6: TNFα-stimulated gene-6
