# Supplementary material for: Evaluation of neuroprotective and immunomodulatory properties of mesenchymal stem cells in an ex vivo retinal explant model
Source: J Neuroinflammation. 2022 Mar 2;19:63. doi: 10.1186/s12974-022-02418-w (PMC8892697; doi:10.1186/s12974-022-02418-w)
Supplement: Supplementary file 1 — Additional file 1. Flow cytometric images showing the histogram overlays of specific antibodies vs isotypic-matched immunoglobulins for each marker from one BMMSC production batch with table presenting the percentages of positive cells and means of fluorescence intensities (MFI) for each marker and each production, with their means, and standard error of the means (SEM). [file 12974_2022_2418_MOESM1_ESM.docx]

**
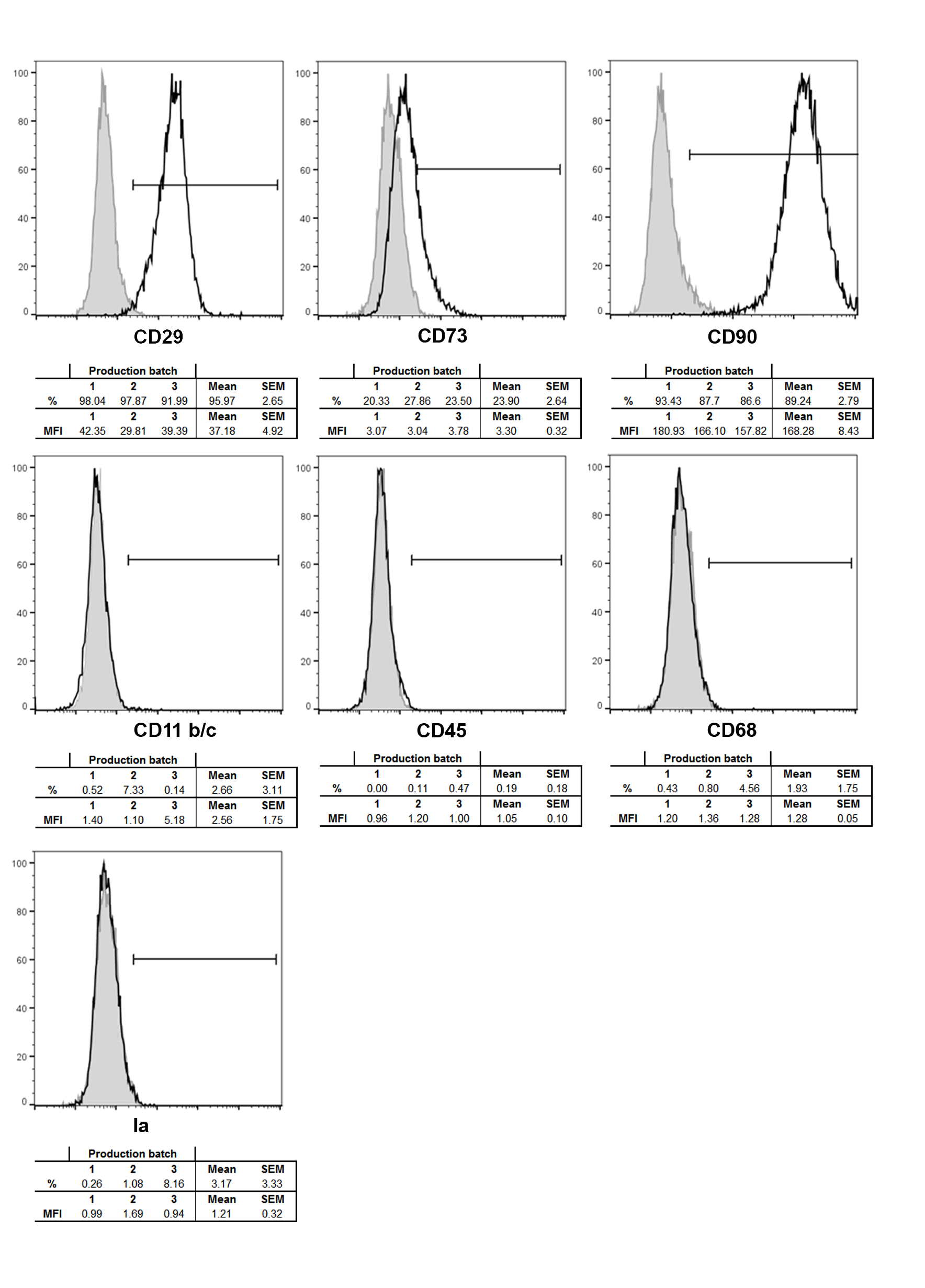
**

**Additional File 1.** Flow cytometric images showing the histogram overlays of specific antibodies vs isotypic-matched immunoglobulins for each marker from one BMMSC production batch with table presenting the percentages of positive cells and means of fluorescence intensities (MFI) for each marker and each production, with their means, and standard error of the means (SEM).
