# Supplementary material for: Evaluation of neuroprotective and immunomodulatory properties of mesenchymal stem cells in an ex vivo retinal explant model
Source: J Neuroinflammation. 2022 Mar 2;19:63. doi: 10.1186/s12974-022-02418-w (PMC8892697; doi:10.1186/s12974-022-02418-w)
Supplement: Supplementary file 2 — Additional file 2. A Representative images of retinal explant cryosections cocultured with 1.104 MSCs for 7 days at DEV 0 and DEV 7, immunolabeled with ChAT (red) and DiO labeled MSCs (green) at ×200 magnification (scale bar = 100 µm). B Quantification of ChAT+ DACs from retinal explants (n = 4–6/day) co-cultured with 1.104 MSCs for 7 days. DACs counts on cryosections are expressed as DACs/mm. [file 12974_2022_2418_MOESM2_ESM.docx]

**
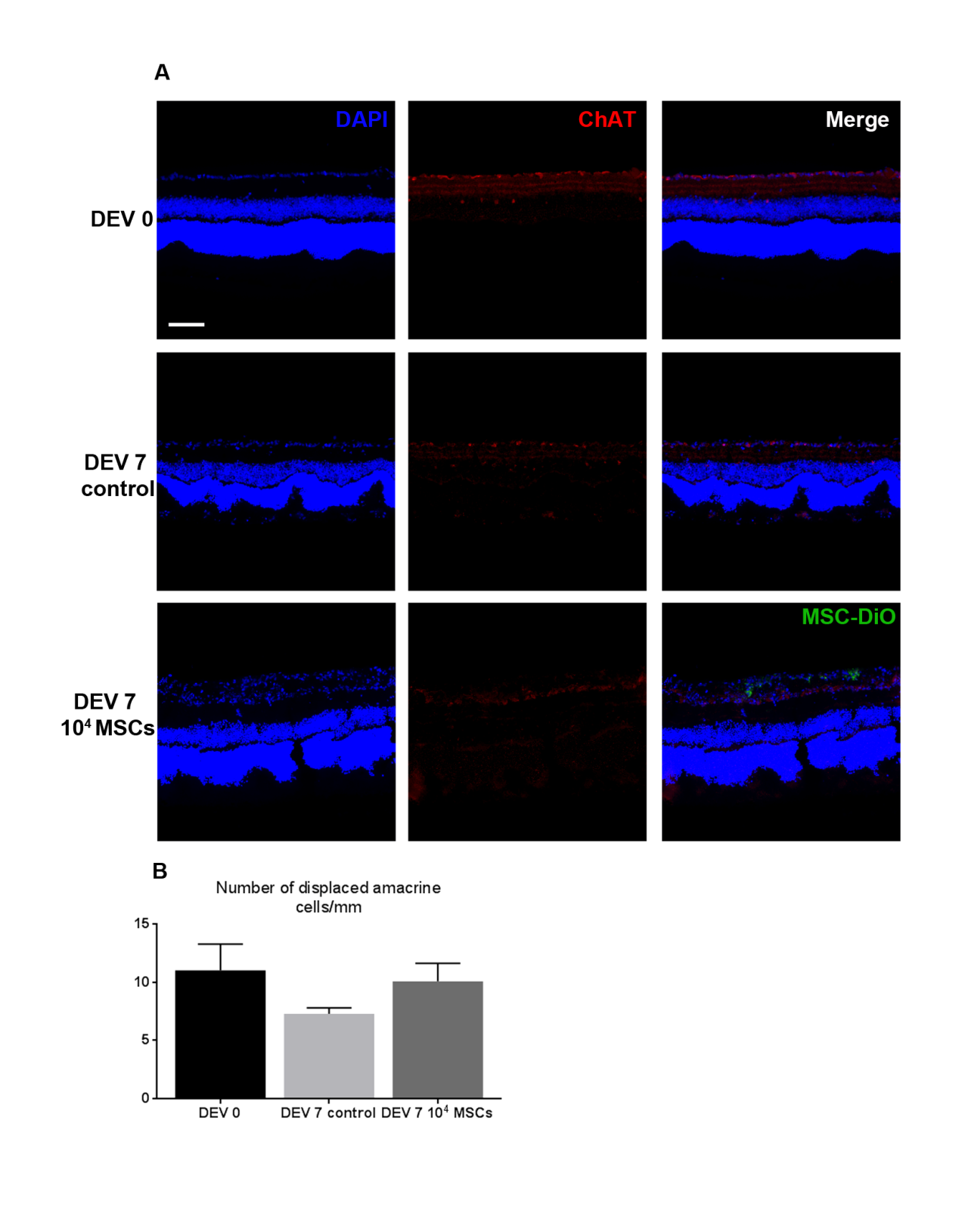
Additional File 2. A**. Representative images of retinal explant cryosections cocultured with 1.10^4^ MSCs for 7 days at DEV 0 and DEV 7, immunolabeled with ChAT (red) and DiO labeled MSCs (green) at 200x magnification (scale bar = 100 µm). **B.** Quantification of ChAT+ DACs from retinal explants (n=4-6/day) co-cultured with 1.10^4^ MSCs for 7 days. DACs counts on cryosections are expressed as DACs/mm.
